# Growth‐regulating proteins differ between British seawater fish species, shedding light on their ecological adaptations

**DOI:** 10.1111/jfb.70323

**Published:** 2026-01-09

**Authors:** Angeliki Maravelia, Soniya Malik, Clare Murcott, Ioannis Batzakas, Paraskevi Goggolidou

**Affiliations:** ^1^ Department of Marine Sciences University of the Aegean Mytilene Greece; ^2^ Faculty of Science and Engineering, School of Pharmacy and Life Sciences University of Wolverhampton Wolverhampton UK

**Keywords:** *Chelidonichthys cuculus*, *Microstomus kitt*, *Pleuronectes platessa*, *Scomber scombrus*, Wnt signalling

## Abstract

Wnt proteins are a family of molecules that help control how cells grow, develop and communicate – processes that are fundamental to the development and health of all animals. Although Wnt pathways have been studied extensively in model species, very little is known about how they operate in marine fish. Understanding these pathways in fish may provide important insights into their physiology, development and ecological adaptations. In this study, we examined whether key Wnt‐related proteins are present in four common British seawater fish species – mackerel (*Scomber scombrus*), plaice (*Pleuronectes platessa*), lemon sole (*Microstomus kitt*) and red gurnard (*Chelidonichthys cuculus*) – none of which have previously been investigated for Wnt expression. Using tissue sectioning and immunostaining, clear species‐specific patterns were identified. *S. scombrus* showed expression of Wnt3, Wnt10a, Dvl1, Dvl3 and E‐cadherin. *P. platessa* exhibited stronger expression of Wnt5a and Wnt11, with only low levels of Dvl proteins. Wnt5a was also detected in both *M. kitt* and *C. cuculus*, whereas *M. kitt* additionally expressed Wnt11, Dvl1 and Dvl3. Very little Dvl3 signal was observed in *C. cuculus*. These results provide the first evidence that Wnt‐related proteins are present in these British fish species and that their expression patterns differ significantly among them. This variation may reflect species‐specific developmental or ecological traits, and it lays the groundwork for future studies on the functional significance of Wnt signalling in marine fish biology.

## 1 INTRODUCTION

Wnt signalling is a complicated and intensely studied protein network that is important for cell proliferation, cell migration, cell polarity and determination of cell fate during embryonic development (MacDonald et al., 2009). Wnt proteins are secreted by glycoproteins, consisting of ~350–400 amino acids each, including 22–24 conserved cysteine residues (Takada et al., 2017). To date, 19 isolated Wnt proteins have been identified, with each having a different individual function. In addition, there are numerous other identified and possibly unidentified proteins that play a part in the Wnt signalling pathway, highlighting the complicated network of interactions and functions of the pathway. The Wnt proteins themselves are known to bind and activate the Frizzled (Fz) receptor family, a seven‐transmembrane‐spanning protein family (Komiya & Habas, 2008). Binding of the Wnt ligand and Fz receptor occurs specifically at the N‐terminal cysteine‐rich domain and may require the action of co‐receptors to facilitate Wnt signalling (Komiya & Habas, 2008). This binding can result in the activation of one of the three known Wnt signalling pathways.

## 2 WNT SIGNALLING

The Wnt signalling network consists of two major branches: the canonical (β‐catenin‐dependent) pathway and the non‐canonical (β‐catenin‐independent) pathway, the latter comprising the planar cell polarity (PCP) and calcium (Ca^2+^) pathways (Lancaster & Gleeson, 2010). Although activated by similar ligand–receptor interactions, these pathways regulate different cellular processes. The canonical pathway controls gene expression by stabilising β‐catenin, whereas the PCP pathway regulates cytoskeletal organisation and cell movement and the Ca^2+^ pathway modulates intracellular calcium dynamics.

In canonical signalling, ligands such as Wnt1, Wnt2, Wnt8 and Wnt10a bind to Frizzled (Fz) receptors and LRP5/6 co‐receptors (Goggolidou, 2014). This prevents the breakdown of β‐catenin by the destruction complex (Stamos & Weis, 2013) and activates Dishevelled (Dvl), allowing β‐catenin to enter the nucleus and drive the expression of genes involved in growth and proliferation (Kjolby & Harland, 2017).

The non‐canonical PCP pathway, activated by ligands such as Wnt6, Wnt7 and Wnt11, is key in shaping tissues during early development, including well‐documented roles in convergent extension in zebrafish and *Xenopus* (Komiya & Habas, 2008; Lancaster & Gleeson, 2010). Through Dvl‐mediated activation of RhoA, Rac1 and the associated cytoskeletal regulators, this pathway influences cell polarity, motility and tissue organisation (Simons & Walz, 2006). Some ligands, including Wnt4 and Wnt5a, can activate either canonical or non‐canonical pathways depending on tissue context and are involved in diverse developmental processes (Ackers & Malgor, 2018; Kumawat & Gosens, 2016). These well‐established mechanisms from model organisms provide an essential framework for exploring Wnt signalling in marine fishes, where its developmental, physiological and ecological roles remain largely uncharacterised.

## 3 BRITISH MARINE FISH

Because Wnt signalling plays such a diverse role, in this study, we sought to identify the presence of any Wnt‐related proteins to determine whether the Wnt signalling pathway is expressed in four different types of fish in which no previous data have been published. The fish tissues investigated in this study are *Chelidonichthys cuculus* (red gurnard), *Scomber scombrus* (British mackerel), *Microstomus kitt* (lemon sole) and *Pleuronectes platessa* (British whole plaice).

The four fish species selected for this investigation were chosen to encompass a range of phylogenetically and ecologically distinct teleosts, commonly found in British waters. *C. cuculus* is a demersal scorpaeniform fish widely distributed in the Northeast Atlantic and Mediterranean, found from shallow coastal grounds to depths exceeding 300 m (Froese & Pauly, 2024). It is characterised by distinctive pectoral fin rays used for benthic locomotion and prey detection. As an opportunistic predator of crustaceans and small fish, it occupies an important trophic position. Its complex fin morphology and benthic adaptations make it a useful species for investigating structural and developmental signalling pathways. *S. scombrus* is a fast‐swimming pelagic species with high metabolic turnover and specialised red and white muscle distribution supporting sustained cruising behaviour (Wardle & He, 1988). It forms large migratory schools throughout the North Atlantic and plays a major ecological role as both predator and prey. Its contrasting physiology and life history relative to benthic species provide a valuable comparison for assessing Wnt expression in a pelagic, high‐performance teleost. On the contrary. *M. kitt* is a right‐eyed pleuronectid flatfish that inhabits sandy and mixed substrates from 20 to 200 m depth (Froese & Pauly, 2024). Like other flatfishes, it undergoes dramatic metamorphosis during the larval–juvenile transition, including eye migration, cranial restructuring and changes in pigmentation (Bolker & Hill, 2000). These developmental processes are known to depend on tightly regulated cell signalling cascades, making flatfishes ideal candidates for investigating signalling pathways such as Wnt. *P. platessa* is one of the most extensively studied flatfish species in the Northeast Atlantic due to its commercial significance and well‐described life history (Rijnsdorp & van Leeuwen, 1992). It exhibits significant seasonal migrations, strong environmental sensitivity during juvenile settlement and pronounced asymmetry after metamorphosis (Nash & Geffen, 2014). Its well‐characterised developmental biology provides an essential reference point for interpreting molecular signals in other flatfishes.

This selection enables the assessment of Wnt pathway expression across species with differing morphologies, habitats and life histories, thereby providing a comparative perspective on the potential conservation of Wnt‐related signalling components. Furthermore, as no previous studies have characterised Wnt signalling in these species, this work contributes novel baseline data with both biological and ecological relevance, while also offering insight into the broader evolutionary conservation of Wnt‐mediated processes in marine fishes.

## 4 MATERIALS AND METHODS

### 4.1 Ethics statement

No live animals were used in this study. All fish samples were obtained as commercially sourced food products from a UK supermarket. As the work did not involve live animals and no regulated procedures were performed, approval under the Animals (Scientific Procedures) Act 1986 was not required.

### 4.2 Fish

The selected fish types were identified as *C. cuculus* (red gurnard), *S. scombrus* (British mackerel), *M. kitt* (lemon sole) and *P. platessa* (British whole plaice). Initially, the head, fins and tail were removed, whereas the skin remained intact to recognise the dorsal and ventral sides of the tissue. The central bone and smaller bones were removed where possible to avoid any problems in dissection on a microtome. Each side of the fish was divided into three parts, namely closest to the head, in the middle and near the tail.

### 4.3 Tissue preparation

Tissue samples were fixated in paraformaldehyde for 48 h before being sliced into one of three parts: the head, middle or tail. Further dehydration steps were carried out by washing the tissue samples in ethanol, xylene and finally paraffin wax (56–58°C) twice for 1.5 h each. The tissues were then embedded in paraffin blocks and sectioned into thinner slices using a microtome (5–7 μm thick) before being transferred onto positively charged slides.

### 4.4 Immunofluorescence

Immunohistochemistry was performed on tissue sections using the following antibodies: Wnt3 (1:200, SC74537, Santa Cruz), Wnt10a (1:200, SC‐376028, Santa Cruz), Dvl (B‐4) (1:200, SC‐166303, Santa Cruz), Dvl3 (1:200, SC‐271295, Santa Cruz) and E‐cadherin (1:200, 3195S, Cell Signaling). Alexa Fluor Anti‐Mouse 488 (1:500, Ab150113, Abcam) or Alexa Fluor Anti‐Rabbit 594 (1:500, A12381, Invitrogen) was used as the secondary antibody. Staining was visualised using a Zeiss LSM880 confocal microscope, and the intensity of expression was quantified using ImageJ. *C. cuculus* was used as the control tissue whereby treatment differed by the lack of primary antibody treatment (Table 1).

**TABLE 1:** A mean measure showing the intensity of expression of each antibody in the different fish types, namely red gurnard (*Chelidonichthys cuculus*), British mackerel (*Scomber scombrus*), lemon sole (*Microstomus kitt*) and British whole plaice (*Pleuronectes platessa*).

Fish tissue type	Control	Wnt5a	Wnt11	Dvl1	Dvl3
British mackerel	–	–	20.687	32.965	28.012
British whole plaice	–	30.324	29.500	24.521	15.843
Lemon sole	–	26.614	16.694	19.166	19.827
Red gurnard	0.134	23.115	14.168	17.169	18.948

*Note:* A red gurnard fish tissue sample was used as control, which was not treated with a primary antibody.

### 4.5 Statistical methods

Data were analysed using paired *t*‐tests, with significance being accepted at *p <* 0.05. Error bars in all data represent standard error of mean (*n* = 3).

## 5 RESULTS

### 5.1 Dvl1 and Dvl3 are expressed in *S. scombrus*

**FIGURE 1:**
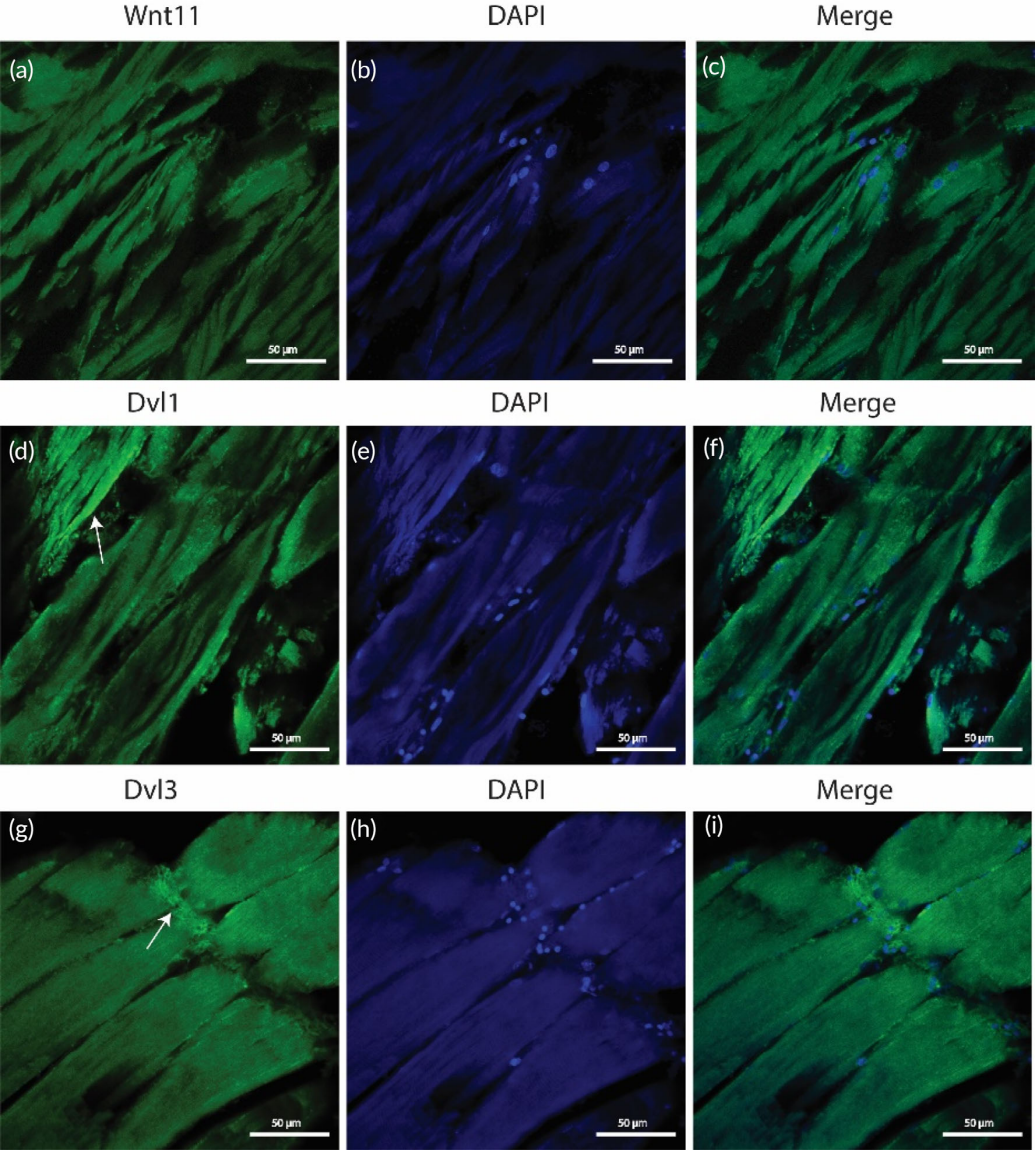
Immunohistochemistry revealing expression of Wnt11, Dvl1 and Dvl3 proteins in British mackerel tissue samples. (a–c) Presence of Wnt11; (d–f) presence of Dvl1; (g–i) presence of Dvl3. Scale bar: 50 μm.

Immunohistochemical analysis of *S. scombrus* tissue exhibited a weak fluorescent signal for Wnt11 (20.687; Figures 1a–c and Table 1). In comparison, strong Dvl1 expression (32.695; Figures 1d–f and Table 1) and moderate but localised Dvl3 expression (28.012; Figures 1g–i, white arrow, and Table 1) were observed. Appropriate control testing was performed to demonstrate that no signal was detected in fish tissues in the absence of primary antibody (Figure S1).

### 5.2 Strong Wnt5a and Wnt11 expression is detected in *P. platessa*

**FIGURE 2:**
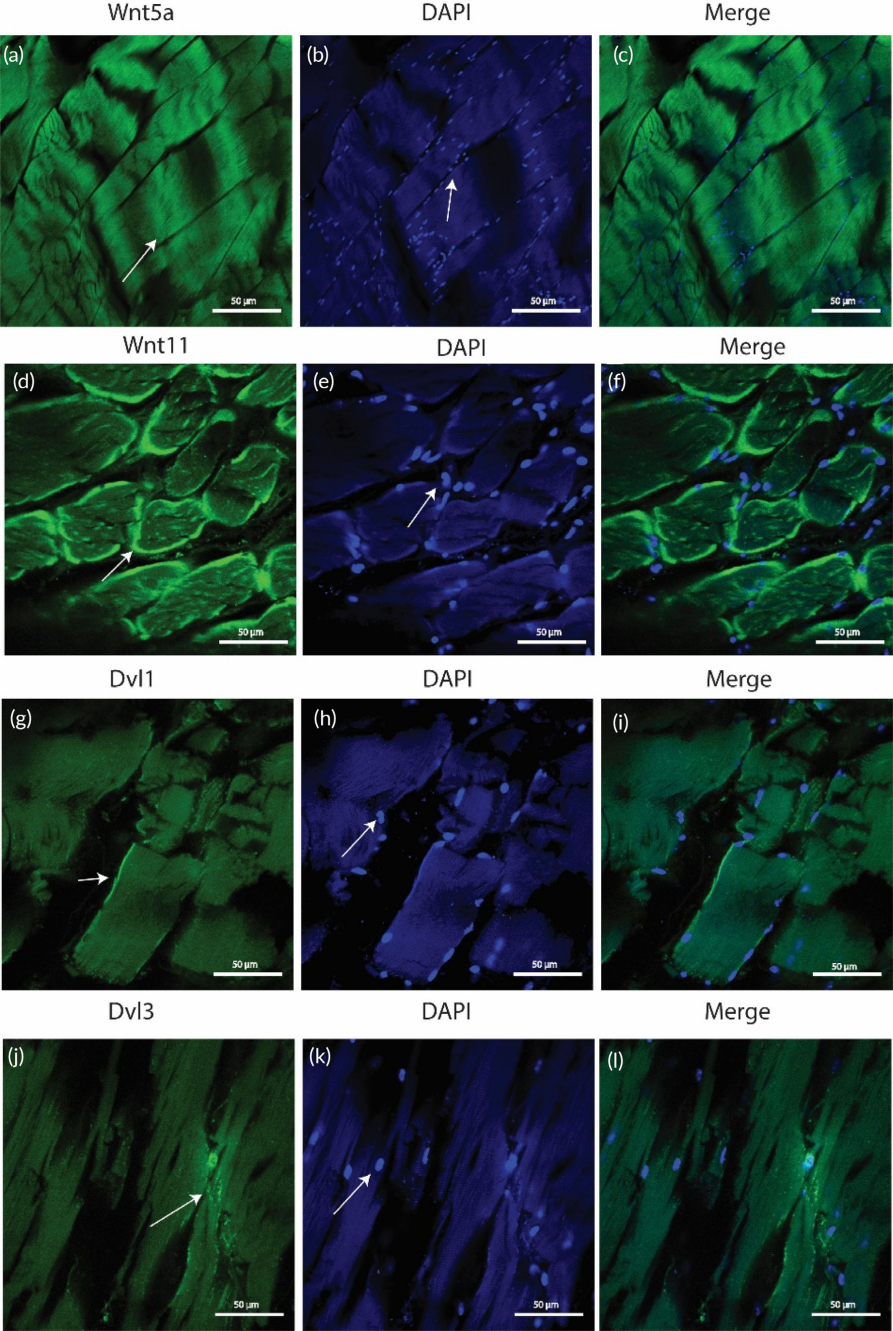
Immunohistochemistry revealing expression of Wnt5a, Wnt11, Dvl1 and Dvl3 proteins in British whole plaice tissue samples. (a–c) Presence of Wnt5a; (d–f) presence of Wnt11; (g–i) presence of Dvl1; (j–l) presence of Dvl3. Scale bar: 50 μm.

Protein expression analysis in *P. platessa* revealed uniform expression of Wnt5a across the tissue (30.324; Figures 2a–c and Table 1). Comparatively, strong Wnt11 expression was detected apically, manifesting a more localised expression pattern (29.500; Figures 2d–f and Table 1). On the contrary, very low Dvl1 (24.521; Figures 2g–i and Table 1) and Dvl3 (15.843; Figures 2j–l and Table 1) expression was observed.

### 5.3 Strong apical Wnt5a expression in *M. kitt* and moderate Wnt5a expression in *C. cuculus* are observed

**FIGURE 3:**
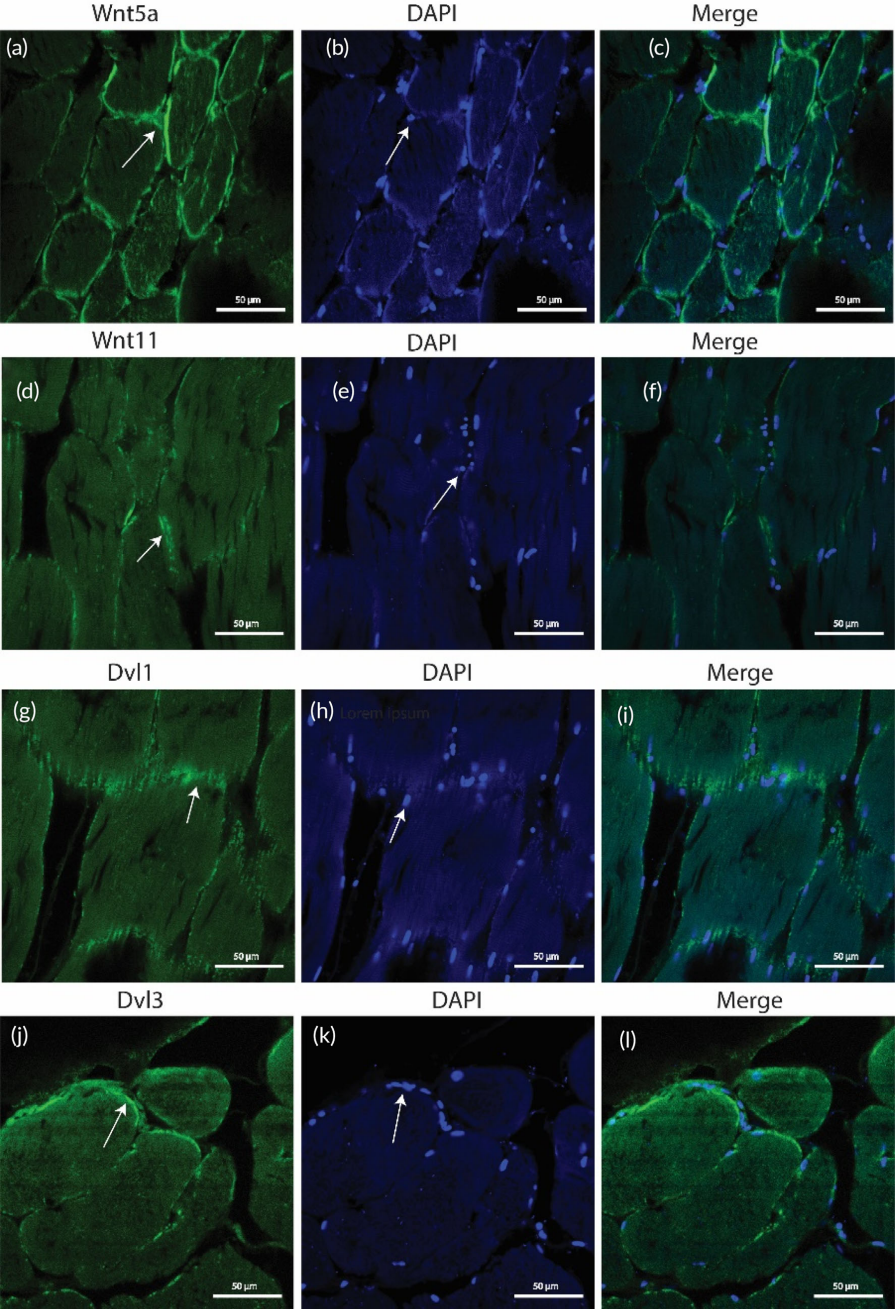
Immunohistochemistry revealing expression of Wnt5a, Wnt11, Dvl1 and Dvl3 proteins in lemon sole tissue samples. (a–c) Presence of Wnt5a; (d–f) presence of Wnt11; (g–i) presence of Dvl1; (j–l) presence of Dvl3. Scale bar: 50 μm.

Immunohistochemical staining for Wnt5a, Wnt11, Dvl1 and Dvl3 in *M. kitt* highlighted a specific expression pattern which was unlike the Wnt signalling protein expression observed in *S. scombrus* and *P. platessa*. Wnt5a exhibited strong apical expression (26.614; Figures 3a–c and Table 1), whereas comparatively, limited localised Wnt11 expression was observed (16.694; Figures 3d–f and Table 1). Dvl1 and Dvl3 also exhibited little, patchy expression, demonstrating that a few cells expressed Dvl proteins in *M. kitt* (19.166 and 19.827, respectively; Figures 3g–l and Table 1).

**FIGURE 4:**
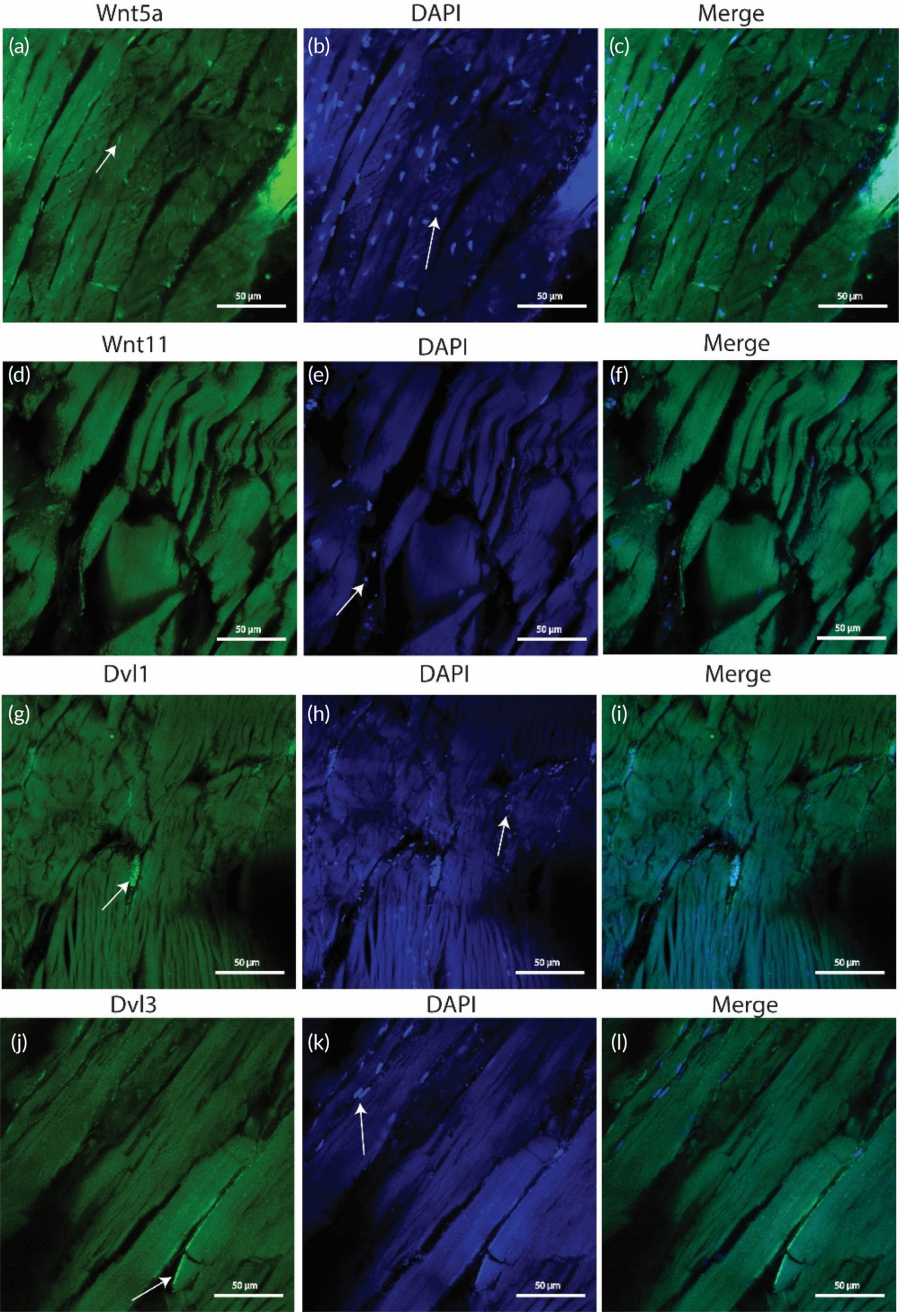
Immunohistochemistry revealing expression of Wnt5a, Wnt11, Dvl1 and Dvl3 proteins in red gurnard tissue samples. (a–c) Presence of Wnt5a; (d–f) presence of Wnt11; (g–i) presence of Dvl1; (j–l) presence of Dvl3. Scale bar: 50 μm.

Moderate, localised Wnt5a expression was observed in *C. cuculus* (23.115; Figures 4a–c, white arrow, and Table 1). Dispersed, weak Wnt11 expression was also recorded (14.168; Figures 4d–f and Table 1) throughout the tissue, leading to moderate, localised expression of Dvl1 (17.169; Figures 4g–i, white arrow, and Table 1) and Dvl3 (18.948; Figures 4j–l, white arrow, and Table 1). Overall, low expression of Wnt signalling proteins was detected in *C. cuculus* (Figure 7).

### 5.4 Non‐canonical Wnt signalling proteins demonstrated strong expression in *S. scombrus*

**FIGURE 5:**
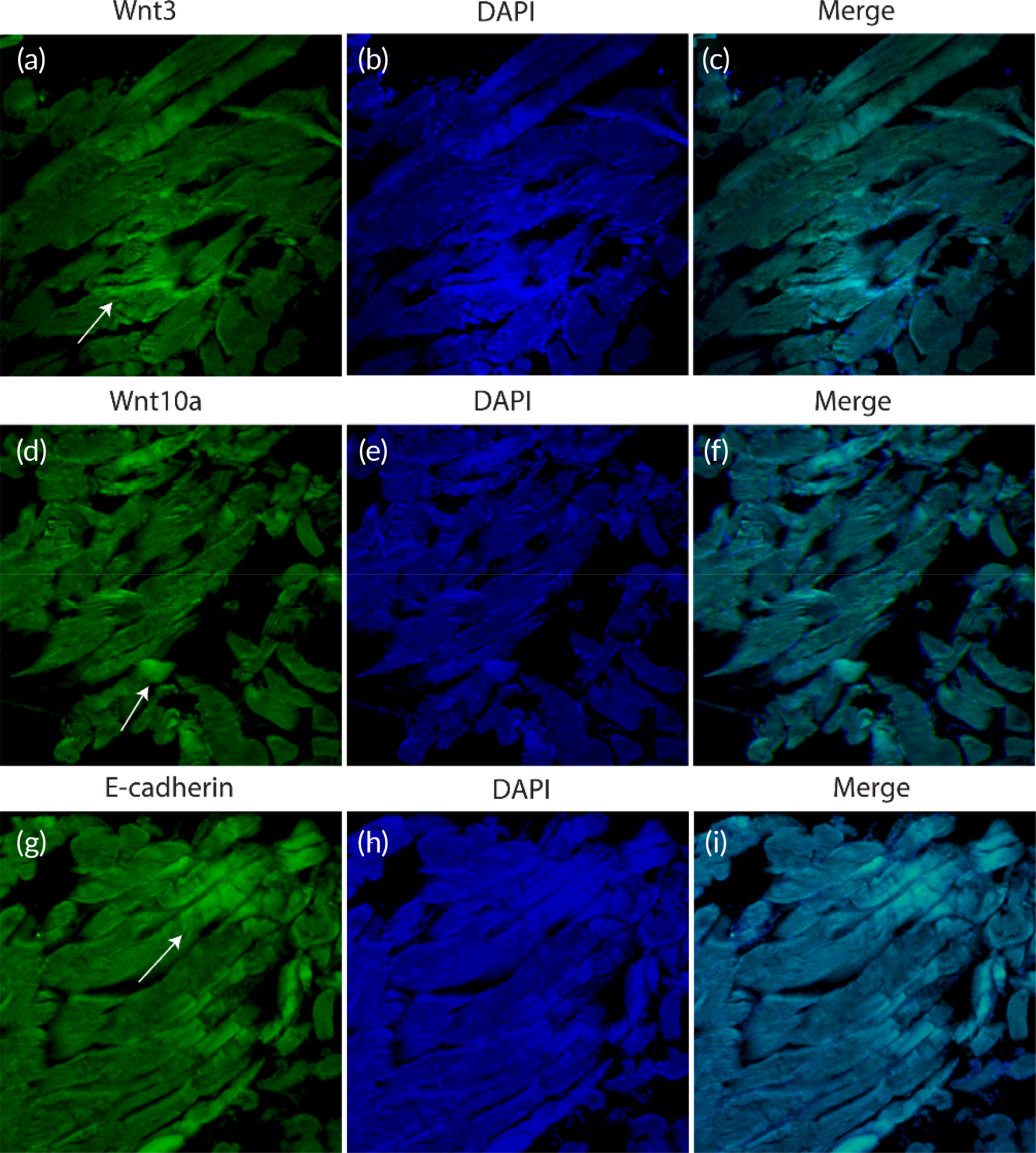
Three dimensional z stack confocal microscopy revealing expression of Wnt3, Wnt10a and E‐cadherin proteins in British mackerel tissue samples. (a–c) Presence of Wnt3; (d–f) presence of Wnt10a; (g–i) presence of E‐cadherin. Scale bar: 100 μm.

Further immunohistochemical analysis of proteins expressed in *S. scombrus* revealed strong Wnt3, Wnt10a and E‐cadherin expression (Figure 5a‐c). Despite our earlier work that exhibited a weak Wnt11 signal (Figure 1a–c) in *S. scombrus*, our further investigations exhibited moderate to strong Wnt3a and Wnt10a expression (Figure 5d‐f). Strong E‐cadherin expression was detected in this fish type (Figure 5g‐i).

### 5.5 Dvl expression was detected in *P. platessa*

**FIGURE 6:**
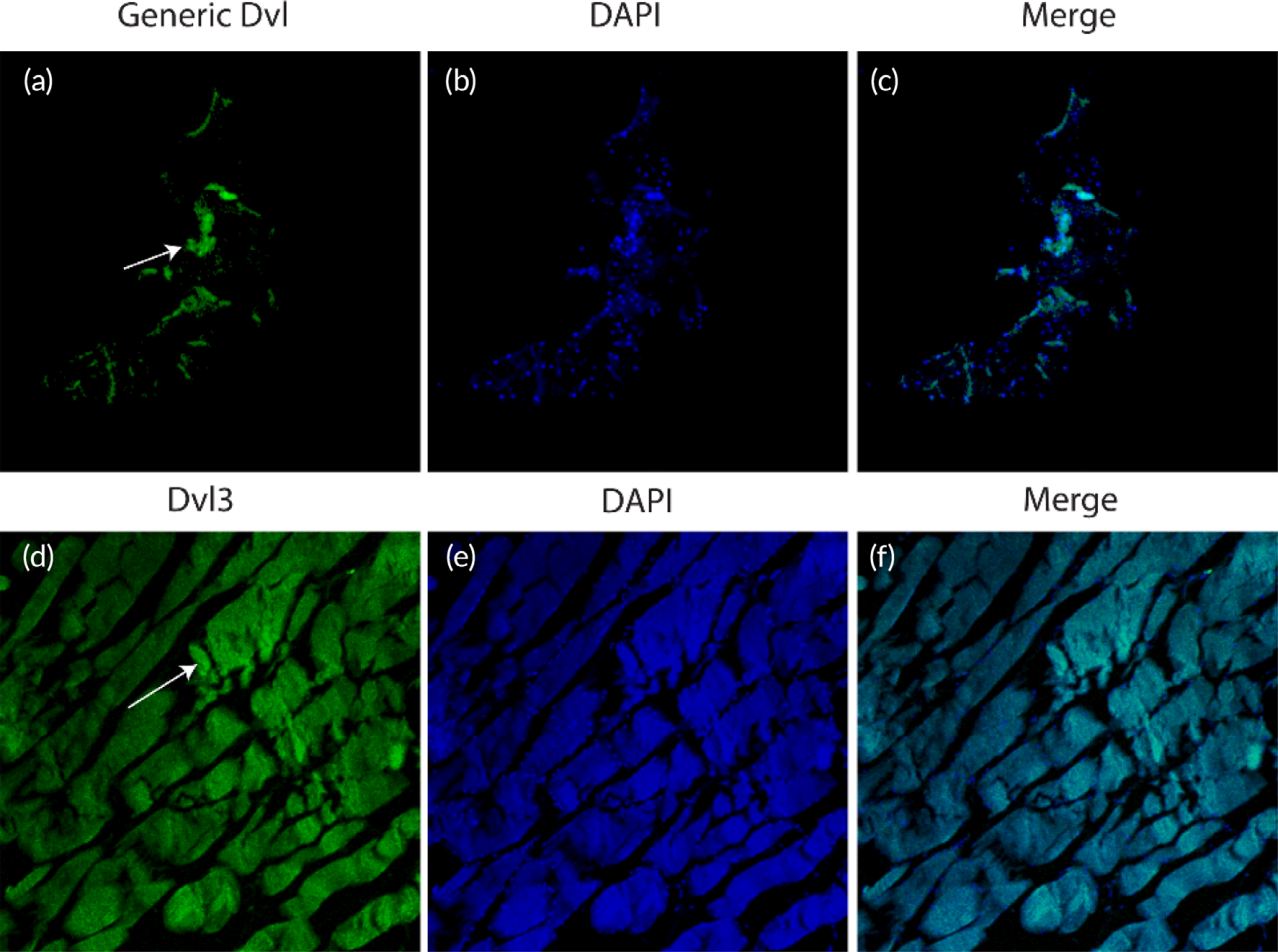
Immunohistochemistry revealing expression of generic Dvl and Dvl3 in British whole plaice tissue samples. (a–c) Presence of generic Dvl; (d–f) presence of Dvl3. Scale bar: 100 μm.

**FIGURE 7:**
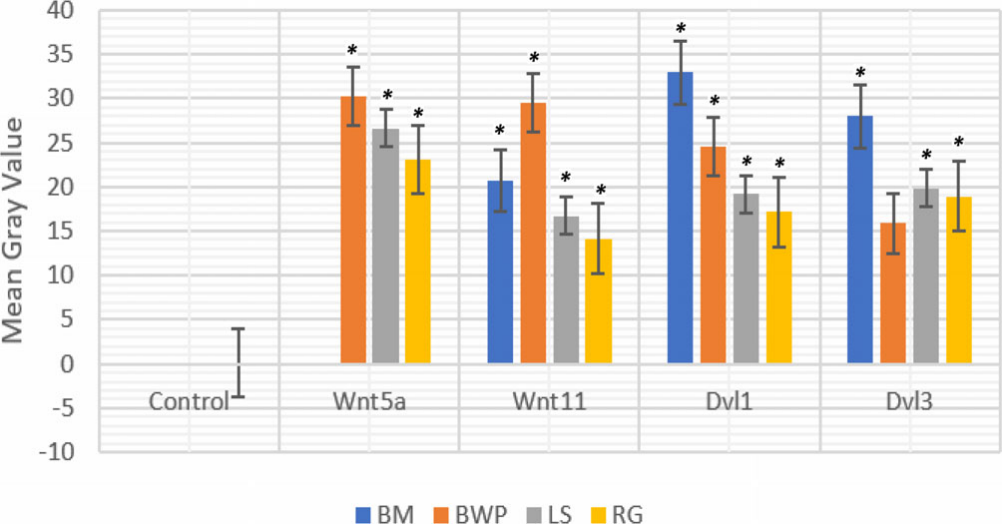
Bar chart plotting mean measure intensity of each protein expression for the four fish types studied. Error bars represent standard error of mean, stars signify *p* value < 0.05). BM: british mackerel; BWP: british whole plaice; LS: lemon sole; RG: red gurnard.

Although strong Wnt5a and Wnt11 expression was observed in *P. platessa*, no Dvl1 expression was detected (Figure 2g–i). Interestingly, Wnt proteins lead to the activation of Dvl, and thus, further analysis was conducted to identify whether there is expression of any other Dvl homologues (Figure 7). Z‐stack imaging of *P. platessa* revealed strong, localised Dvl expression whereby generic Dvl protein was highlighted (Figure 6a‐c), also correlating to the previous Dvl1 staining pattern (Figure 2g–i). Similarly, strong Dvl3 expression was also observed throughout the tissue (Figure 6d‐f), signifying that Wnt5a and Wnt11 expression likely results in Dvl3 activation in *P. platessa*.

## 6 DISCUSSION

This study aimed to investigate whether any expression of Wnt signalling proteins was present in four different fish types, namely *C. cuculus, S. scombrus, M. kitt* and *P. platessa*. Investigation showed that the Wnt signalling pathway is active in these species, with the detection of several upstream components being highlighted via our staining analysis. *C. cuculus* presented an overall weaker Wnt signalling protein staining pattern, although still highlighting moderate yet localised Wnt5a expression and very weak Wnt11 expression. Because Wnt ligands act upstream of Dvl, as expected, there was moderate expression of both Dvl1 and Dvl3. In *S. scombrus*, cytoskeletal expression patterns can be detected with strong expression of upstream Wnt ligands Wnt3a and Wnt10a; moderate, localised Dvl1 and Dvl3 expression; and strong, downstream E‐cadherin expression. Comparatively, in *M. kitt*, there was strong, apical Wnt5a expression with weaker yet localised Wnt11 expression. There was also some Dvl1 and Dvl3 expression. In *P. platessa*, Wnt5a signal was detected across the tissue, with Wnt11 signal being more apical and localised. Strong Dvl3 expression and generic Dvl expression were also detected. This study is the first to discover that the Wnt signalling pathway is activated in these British fish types.

Several human disorders such as cancers, degenerative disorders and ciliopathies have been associated with aberrant Wnt signalling as revealed in human, animal and cell line experiments (Goggolidou, 2014; Hartmann, 2006; Liang et al., 2003). One of the most extensively studied models for Wnt signalling is in zebrafish. In the 1990s, it was established that the canonical Wnt signalling pathway is required for neural crest (NC) development, with the zebrafish model soon emerging as a key tool for study in NC development and vertebrate gastrulation (Eisen & Weston, 1993; Lekven et al., 2001; Lewis et al., 2004; Sutton et al., 2021). It was later discovered that specific Wnt ligands, such as wnt11 and wnt5a, are required for correct convergent extension movements during zebrafish gastrulation, with wnt5a also playing a role in embryonic development (Heisenberg et al., 2000; Kilian et al., 2003). In the kidneys, wnt5a knockdown in zebrafish was found to result in glomerular cyst formation and dilated renal tubules, implicating Wnt5a in polycystic kidney disease and Robinow syndrome (Huang et al., 2014). More recently, zebrafish models have been vital in identifying a role for Dvl isoforms in Wnt signalling activation during early developmental stages. Dysregulation of *dvl* genes has revealed atypical embryonic axis patterning during gastrulation, with dvl isoform abnormalities in zebrafish exhibiting aberrant convergent extension patterns (Shi, 2020; Xing et al., 2018).

Zebrafish studies have been key for identifying the role of Wnt signalling during embryogenesis and other early developmental events that occur. This has later been translated to human disorders and has been important in understanding their molecular mechanisms. This establishes the benefits of using a smaller and cheaper model for animal studies in vertebrate developmental biology. Due to easy manipulation, common genetic background with humans and simplicity in anatomy, fish provide a system for conducting investigative studies (Jussila & Ciruna, 2017).

Although some evidence implicates certain Wnt ligands in both the canonical and non‐canonical branches of the Wnt signalling pathway, it is largely understood that many Wnt ligands are associated with either branch of the Wnt signalling pathway. Having studied the expression of several upstream Wnt signalling components in this study, it would be beneficial to assess the expression patterns of some downstream components such as β‐catenin (phosphorylated and/or non‐phosphorylated), scribble and Vangl2. This could help further elucidate the Wnt signalling expression patterns in these fish. In addition, because the Wnt signalling pathway is associated with several human disorders, it would be beneficial to extend this analysis to specific organs, for example, the kidneys, to better understand whether these fish could be used for further experimental analysis.

In summary, this study provides the first evidence of Wnt signalling pathway activation in *C. cuculus*, *S. scombrus*, *M. kitt* and *P. platessa*, thereby expanding the current understanding of Wnt‐related protein expression across marine teleosts, in which this pathway has not been previously characterised. The detection of multiple upstream Wnt ligands and associated Dishevelled isoforms across all four species indicates that the key components of both canonical and non‐canonical Wnt pathways are conserved and potentially functional within these taxa. These findings not only underscore the evolutionary conservation of Wnt signalling across diverse vertebrate lineages but also establish these British fish species as novel candidates for future comparative and translational studies. Given the central role of Wnt signalling in embryogenesis, tissue homeostasis and the pathogenesis of human disorders, the identification of pathway activity in these species lays important groundwork for developing alternative marine fish models in vertebrate developmental and disease research. Continued investigation into downstream effectors and tissue‐specific expression will further elucidate the extent of Wnt pathway conservation and its biological significance in non‐model teleosts.

## AUTHOR CONTRIBUTIONS

Angeliki Maravelia generated and analysed the data and helped in manuscript preparation. Soniya Malik analysed the data and wrote the manuscript. Clare Murcott assisted with data acquisition and analysis. Ioannis Batzakas contributed to the initial hypothesis and data generation and edited the manuscript. Paraskevi Goggolidou developed the concept, assisted with data analysis and helped prepare and edit the manuscript.

## FUNDING INFORMATION

None.

## Supporting information


**Figure S1.** Red Gurnard tissue after immunohistochemistry without any primary antibody. Image used as control. Scale bar: 50 μm.

## Data Availability

The data that support the findings of this study are available from the corresponding author upon reasonable request.
