# Supplementary material for: Growth‐regulating proteins differ between British seawater fish species, shedding light on their ecological adaptations
Source: J Fish Biol. 2026 Jan 9;108(6):1701–11. doi: 10.1111/jfb.70323 (PMC13357356; doi:10.1111/jfb.70323)
Supplement: Supplementary file 1 — Figure S1. Red Gurnard tissue after immunohistochemistry without any primary antibody. Image used as control. Scale bar: 50 μm. [file JFB-108-1701-s001.docx]

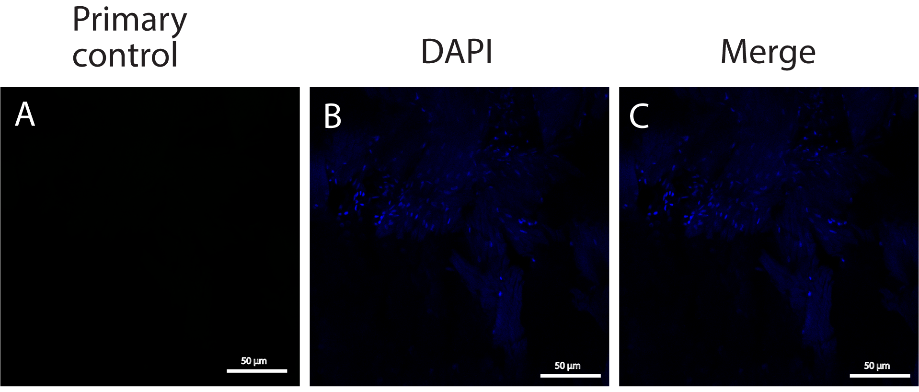


***Supplementary figure S1: Red Gurnard tissue after immunohistochemistry without any primary antibody.*** *Image used as control.* *Scale bar: 50 µm.*
